# What is it like to be a lizard? Directed attention and the flow of sensory experience in lizards and birds

**DOI:** 10.3389/fpsyg.2024.1424329

**Published:** 2024-12-24

**Authors:** Louis N. Irwin

**Affiliations:** Department of Biological Sciences, University of Texas at El Paso, El Paso, TX, United States

**Keywords:** awareness, ethology, proxy behavior, gaze duration, perception, phenomenology, consciousness

## Abstract

While the content of subjective (personal) experience is inaccessible to external observers, behavioral proxies can frame the nature of that experience and suggest its cognitive requirements. Directed attention is widely recognized as a feature of animal awareness. This descriptive study used the frequency of gaze shifts in lizards and birds as an indicator of the rate at which the animals change the perceptual segmentation of their ongoing experience. Most lizards are solitary, with social interactions limited to territorial defense and mating. Many are sit-and-wait insectivores that intersperse active foraging with long periods of sedentary activity. Others actively seek encounters with prey, either randomly (teiids) or through strategies indicative of intelligent planning (varanids). Birds typically change the direction of their attention five times faster than lizards while displaying more behavioral complexity and variety. A number of interspecies differences among both lizards and birds were observed in this study, consistent with the view that subjective experience varies uniquely across lifestyles, ecology, and phylogeny. These differences constitute variations in the structure of perceptual experience and could serve as probes for investigating neural correlates of animal consciousness.

## 1 Introduction

Fifty years ago, Thomas Nagel (1974) asked, “What is it like to be a bat?” His point was that understanding the nature of experience by a bat (or any being whose sensorimotor and perceptual attributes differ substantially from our own) is not attainable within the constraints of current frameworks and linguistics. At the same time he noted that “mental states are states of the body” and that “Conscious experience… occurs at many levels of animal life, though… it is very difficult to say in general what provides evidence of it.” Yet he goes on to speculate that perhaps “we can pursue a more objective understanding of the mental in its own right [if we] form new concepts and devise a new method….”

In the 50 years since the publication of Nagel’s provocative insight, models of and theories about subjective experience in non-human animals have proliferated (Boly et al., 2013; Farhadi, 2024). Kaufmann (2021) has pointed to experience-specific dimensions of consciousness, typical of a growing literature on the interface between phenomenology and cognitive science (Bayne, 2004). This study in that tradition focuses on behavior as an indicator of the animal’s experience. The fundamental difficulty of accessing personal (subjective) experience by external (objective) observers has been noted (Feinberg and Mallatt, 2018; Gallagher, 1997), but the use of proxy behavior has been invoked as one means of circumventing this barrier (Budolfson et al., 2023; Irwin et al., 2022). A related strategy is the pursuit of the structure of animal experience that is objectively describable by an outside observer and coextensive with the animal’s subjective experience of it.

The logic of proxy behavior is that if the same behavior were displayed by a human, it would likely be associated with a subjective experience (Ehret and Romand, 2022). The nature of the mental activity that an animal exhibiting that behavior would experience would not necessarily be the way a human would experience it, but it would be a phenomenological experience of some sort. Not all behavior is conscious, and a distinction between *selective* attention and consciousness has been noted (Tsuchiya and Koch, 2016), but the function of *directed* attention is to bring into focal awareness specific elements of perceptual experience (Nani et al., 2019). While gaze direction cannot always be associated with conscious awareness (Sato et al., 2016; Yang et al., 2024) in humans, direct gaze is a powerful nonverbal cue that plays an important role in social interactions (Madipakkam et al., 2015), and in laboratory animals changes in head direction in particular have identifiable neural correlates that suggest attention of the animal to its location and direction of movement (Moser et al., 2017). Casey (2007) has argued that in fact every glance is attentional to some degree.

Proxy behaviors have been invoked especially in studies on the possibility of pain and suffering in animals (Crook and Walters, 2011; Lambert et al., 2022; Sneddon, 2019) but they have also included emotional behavior in fish (Beritashvili, 1971), problem solving and play in reptiles (Northcutt, 2013), and tool use in birds (Striedter, 2013), to name a few. I have recently used three proxy behaviors (volitional, interactive, and egocentric) quantified in three different ways (frequency, variety, and dynamism) for each mode to generate metaphorical representations of the different patterns of subjective experience across 12 species, including all the living classes of vertebrates (Irwin, 2024). My results were consistent with the view that subjective experience is heterogeneous, multimodal, and non-linear in extent across the animal kingdom.

The present study was designed to probe in greater detail a facet of experience within two classes of vertebrates – reptiles (lizards, specifically) and birds. One of the simplest and easiest behaviors to measure is directed attention (Moore and Zirnsak, 2017), which is a window into awareness (Madipakkam et al., 2015), and hence experience (Farhadi, 2024). As James (1890) put it, “My experience is what I agree to attend to.” Each time an animal changes the direction of its attention – each glance in a different direction – it changes the contours of its sensory experience. Casey (2007) has argued that the visual glance parcels the flow of the organism’s awareness, providing a perceptual frame of its world over a sliver of time. Assuming that the animal mind involves, at a minimum, attention to a succession of perceptual frames of time, place, and space, it follows that the rate at which it shifts its gaze provides a measure of how fast the animal’s perception of its environment flows through its experience of it. This can then be used as one descriptor of what phenomenology is like for that animal.

Assessing the nature of and rate at which those perceptual frames are processed by different species of lizards and birds is the objective of this study. It seeks to objectify one aspect of the structure of an important behavioral process that is coextensive with the animal’s subjective experience of that process. Primary focus is on the visual gaze, with some attention to sampling of taste and other sensorimotor information as well. This information is offered as a sample of what it is like to be a lizard or a bird in the species in this study.

## 2 Methods

### 2.1 Subjects

The following lizards were observed during April, June and July, at the Denver Museum of Nature and Science, at warm ambient temperature within enclosed terraria, between 9:00 and 11:00:

- Rhinocerus iguana, *Cyclura cornuta*, ~ 0.8 m in snout-vent length (SV).
- Frilled lizard, *Chlamydosaurus kingii*, ~ 60 cm (SV).
- Water monitor, Var*anus salvator*, ~ 1.0 m (SV).
- Green tree monitor, *Varanus prasinus*, ~ 1.0 m (SV).
- Blue tongued skink, *Tiliqua scincoides*, ~50 cm (SV).
- Chukawala, *Sauromalus ater*, ~30 cm (SV).
- Eastern water dragon, *Physignathus lesueurii,* 0.8 m (SV).

The following free-living lizards were observed in the open field at the Indio Mountains Research Station of the University of Texas at El Paso, in Hudspeth County, Texas on 10–12 August, generally at an air temperature of 30–35°C, at various hours:

*Texas earless lizard, *Cophosaurus texanus*, 3–5 cm (SV).
*Collared lizard, *Crotophytus collaris*, 6–8 cm (SV) Checkered whiptail, *Aspidoscelis tesselata*, ~8 cm (SV) – a parthenoform.
*Little striped whiptail, *Aspidoscelis inornata*, ~4 cm (SV).

The following birds were observed at the Denver Zoo, between 0830 and 1,130, during April, July and August, generally at 25–30°C.

*Canadian goose, *Branta canadensis,* ~0.8 m, free-living on outdoor lawn Steller’s sea eagle, *Haliaeetus pelagicus,* ~0.6 m, in outdoor enclosure.
*African penguin, *Sphenicus demersus*, ~0.5 m, in outdoor pool and ledge.
*Saddle-billed stork, *Ephippiorhynchus senegalensis*, ~1.2 m, in open field Green-winged macaw, *Ara chloroptera*, ~0.9 m, in indoor aviary.
*Paradise tanager, *Tangara chilensis*, ~12 cm, in indoor enclosure.
*Yellow-breasted ground dove, *Gallicolumba tristigmata*, ~30 cm, in indoor aviary Lappet-faced vulture, *Torgas tracheliotus*, ~0.8 m, in outdoor enclosure.

Asterisks indicate species for which each observation was on a different individual; otherwise multiple observations were of the same individual but always on different days.

### 2.2 Observations

**Figure 1:**
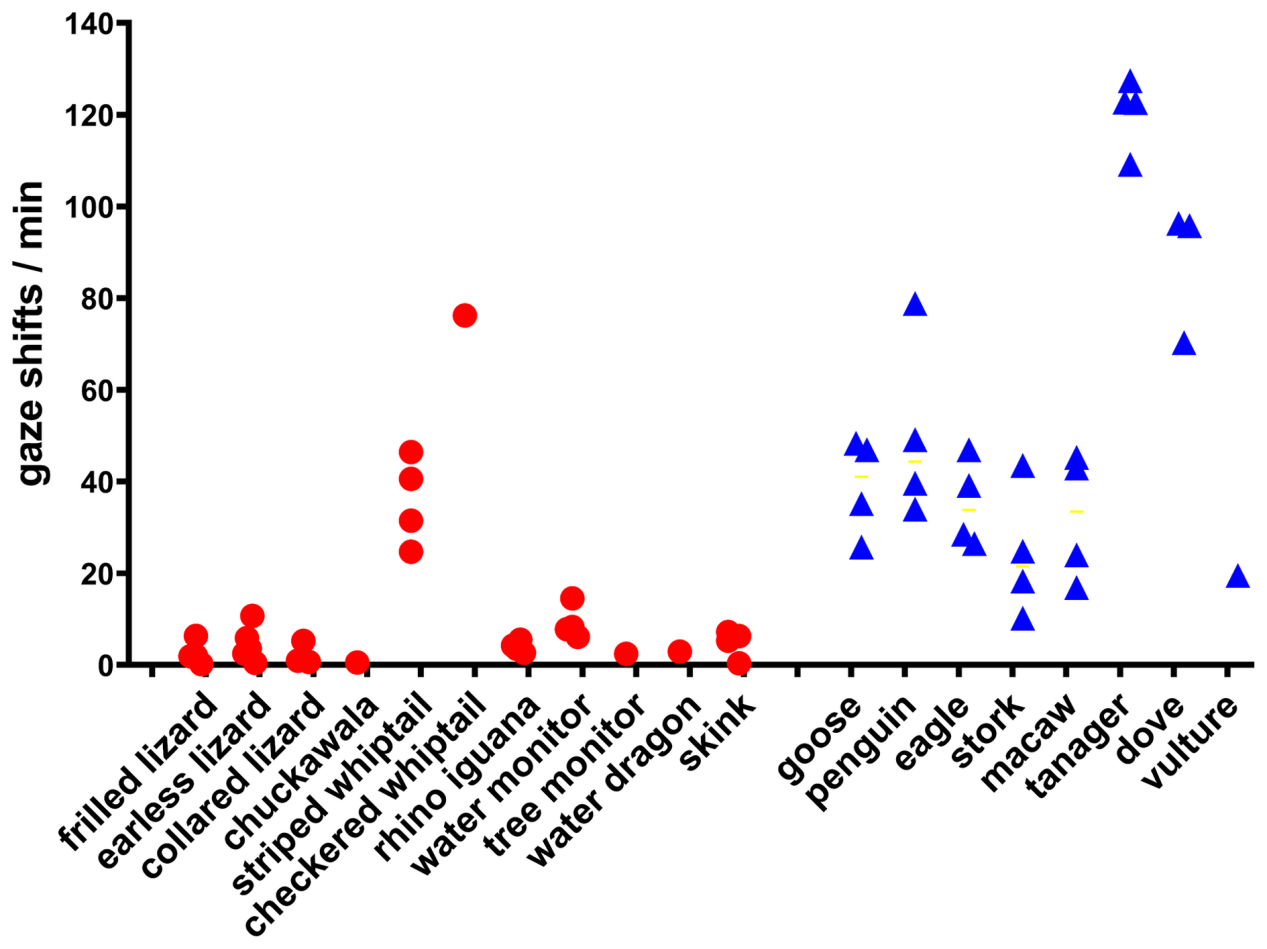
Frequency of gaze shifts for all lizards and birds. Each point is the mean number of gaze shifts/min for every observational session in the study. The mean for all lizards differed significantly from the mean for all birds by Welch’s unpaired 1-tailed *t*-test (*t* = 2.66, df = 11.33, *p* = 0.011).

Most animals were watched in 3–5 separate episodes lasting 10–20 min each (except for *A. inornata,* which was very skittish, active, and hard to keep up with in the field). A few animals were observed only in a single session. These were never included in statistical evaluations, but are shown in Figure 1 and sometimes mentioned in the text where the single observation added useful information.

During each episode, the number of times the animal shifted its visual field, either by moving its eyes or its head, were recorded in 60 s bins. For some species, the number of licks made with the tongue were recorded in parallel to the shifts in gaze.

### 2.3 Statistical analysis

**Figure 2:**
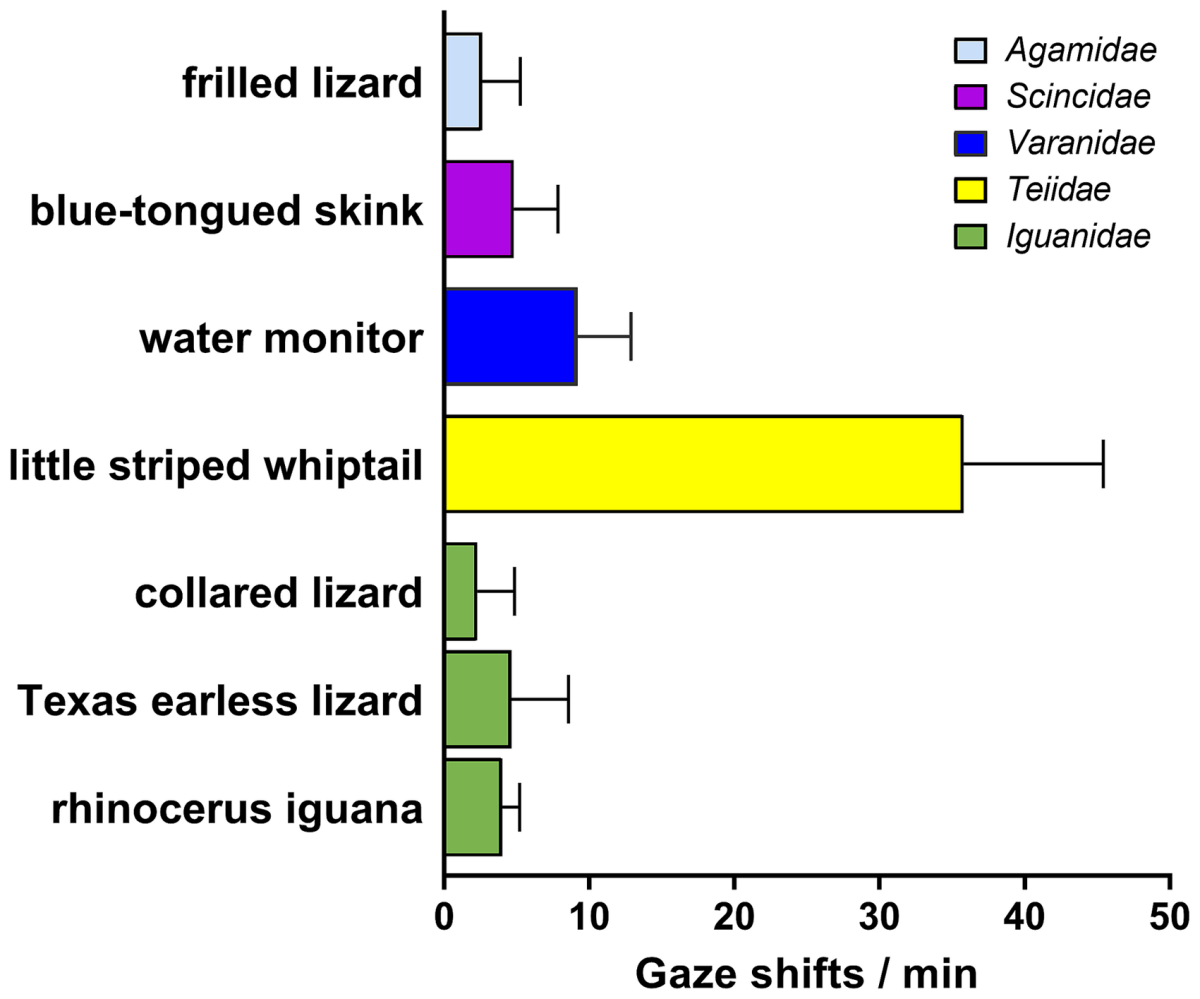
Frequency of gaze shifts for lizards observed in at least three sessions. Bars indicate means + SD, color coded for the taxonomic family or infraorder to which each species belongs. The striped whiptail was a statistical outlier with a probability exceeding 98%, based on the ROUT method of Motulsky and Brown (2006). With it excluded, the remaining species did not vary significantly from one another by a Kruskall-Wallace 1-way ANOVA (KW = 8.62; *n* = 6, *p* > 0.12), but the other clades each differed significantly (*p* < 0.0001) from the striped whiptail by a nested 1-way ANOVA with Dunnett’s correction for multiple comparisons.

**Figure 3:**
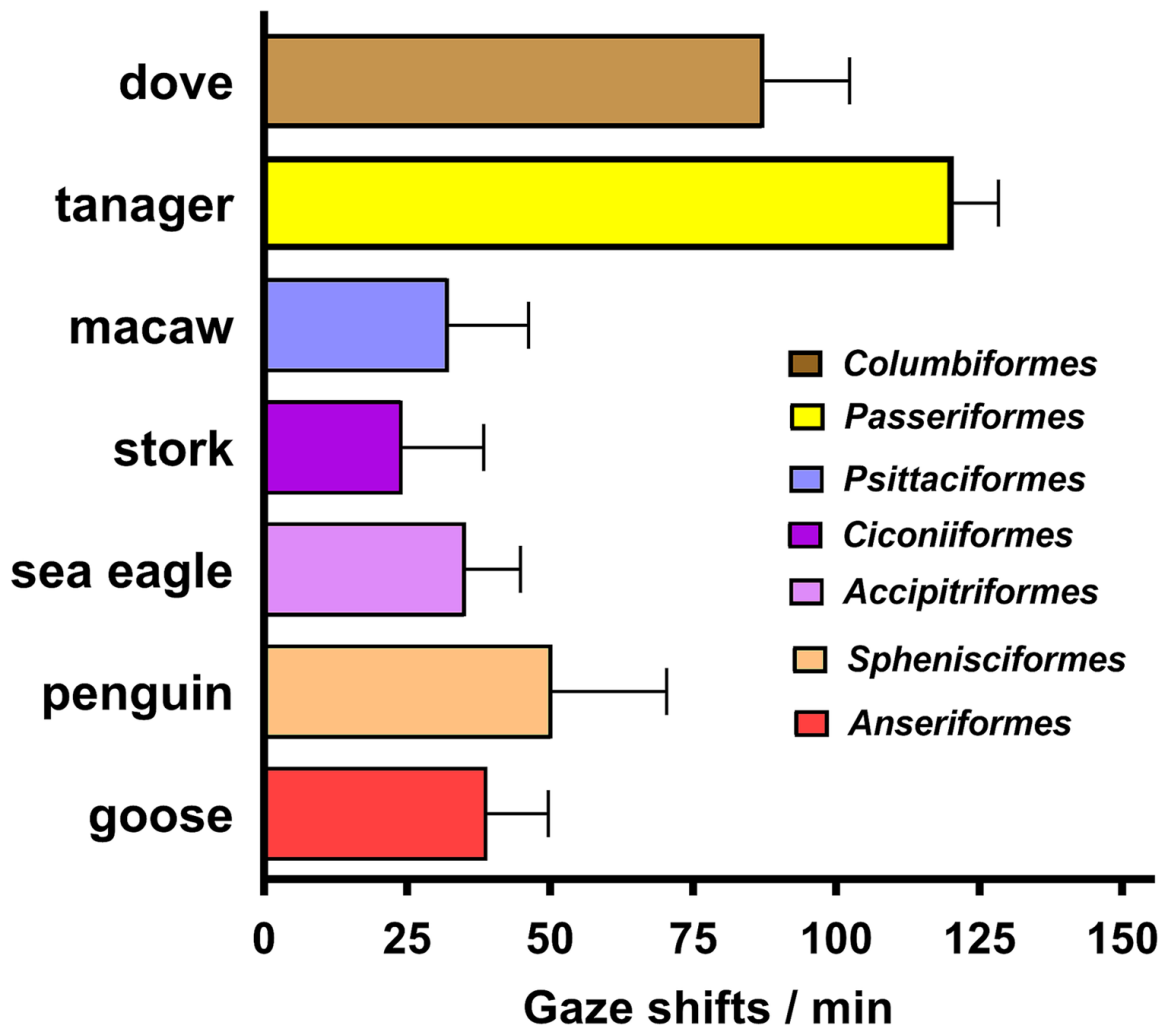
Frequency of gaze shifts for birds observed in at least three sessions. Bars indicate means + SD, color coded for the taxonomic order to which each species belongs. The tanager was a statistical outlier with a probability exceeding 98%, based on the ROUT method of Motulsky and Brown (2006). The high rate of gaze shifts for the dove was not a statistical outlier, but a nested 1-way ANOVA with Dunnett’s correction for multiple comparisons showed it to differ significantly from that of the macaw (*p* = 0.0003), stork (*p* < 0.0001), eagle (*p* = 0.0002), penguin (*p* = 0.012), and goose (*p* = 0.0013).

The difference between lizards and birds as a group in Figure 1 was tested by Welch’s unpaired 1-way *t*-test. The presence of outliers among either the lizards or birds was tested by the ROUT method of Motulsky and Brown (2006). A 1-way Kruskall-Wallace analysis of variance (ANOVA) was used to test whether values across all lizards or birds after exclusion of outliers differed significantly. This non-parametric test was chosen because of the small sample sizes and inability to assume a normal distribution. A nested 1-way ANOVA with Dunnett’s correction for multiple comparisons was used to test the significance of data from the striped whiptail in comparison to that from other lizards in Figure 2, and from the dove in comparison to that from other birds in Figure 3. The statistical software used was embedded in GraphPad Prism, version 9.5.1, and probabilities (exact or less than) are shown as reported by the program.

## 3 Results

The frequency of gaze shifts for all lizard and avian species studied is shown in Figure 1, which includes data from species for which only a single observation was obtained, for the sake of completion. Lizards differ from birds significantly, by Welch’s 1-tailed *t*-test (*t* = 2.66, df = 11.33, *p* = 0.011).

### 3.1 Lizards

Among the lizards for which data were obtained in 3 or more observational sessions (Figure 2), the frequency of gaze shifts/min was 10.6 ± 16.5 (mean ± SD), but the striped whiptail was an outlier at a probability exceeding 98%, so with that species excluded, the frequency falls to 8.4 ± 11.6. This equates to a mean gaze duration of 7.14 s. There were no significant differences across all the species excluding the striped whiptail by a Kruskall-Wallace 1-way ANOVA (*p* > 0.12). However, data from this whiptail differed significantly (*p* < 0.0001) from that for each of the other clades by a 1-way ANOVA with Dunnett’s correction for multiple comparisons.

**Figure 4:**
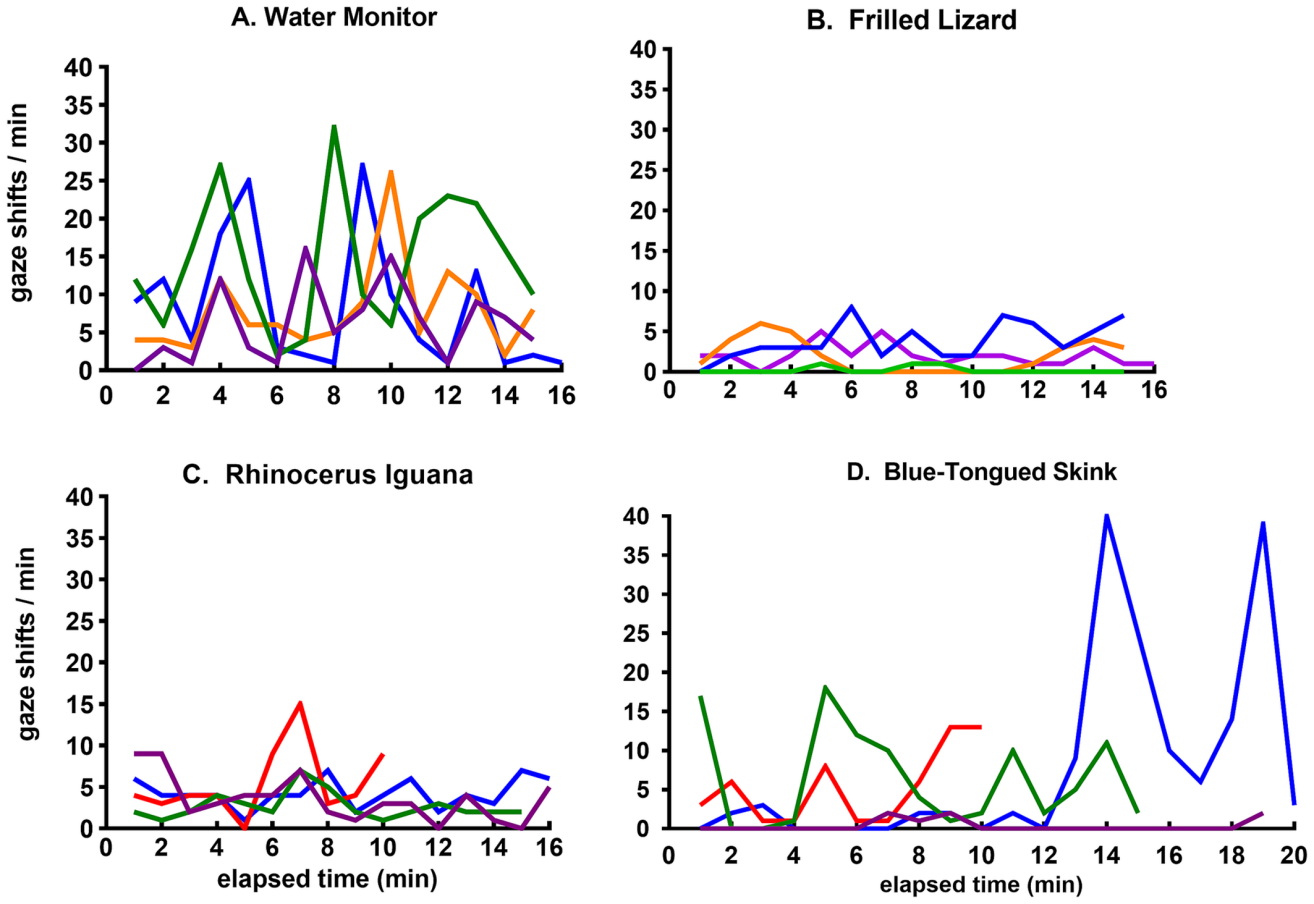
Time course of gaze shifts for the **(A)** water monitor, **(B)** frilled lizard, **(C)** rhinoceros iguana, and **(D)** blue-tongued skink. Each line records minute-to-minute rates of gaze shifts by the same lizard in observational sessions on different days.

A more granular view is provided by plots of minute-to-minute variation in the frequency of gaze shifts for four of the species, each displaying a qualitatively different pattern for the flow of sensory experience (Figure 4). The water monitor showed frequent periods of a high rate of gaze shifts (Figure 4A). The frilled lizard displayed minimal activity with a consistently low rate of gaze shifts (Figure 4B). The rhinoceros iguana showed occasional bursts of frequent gaze shifts superimposed on a generally low level of activity (Figure 4C). The skink alternated between periods of low and high frequency of gaze shifts (Figure 4D).

**Figure 5:**
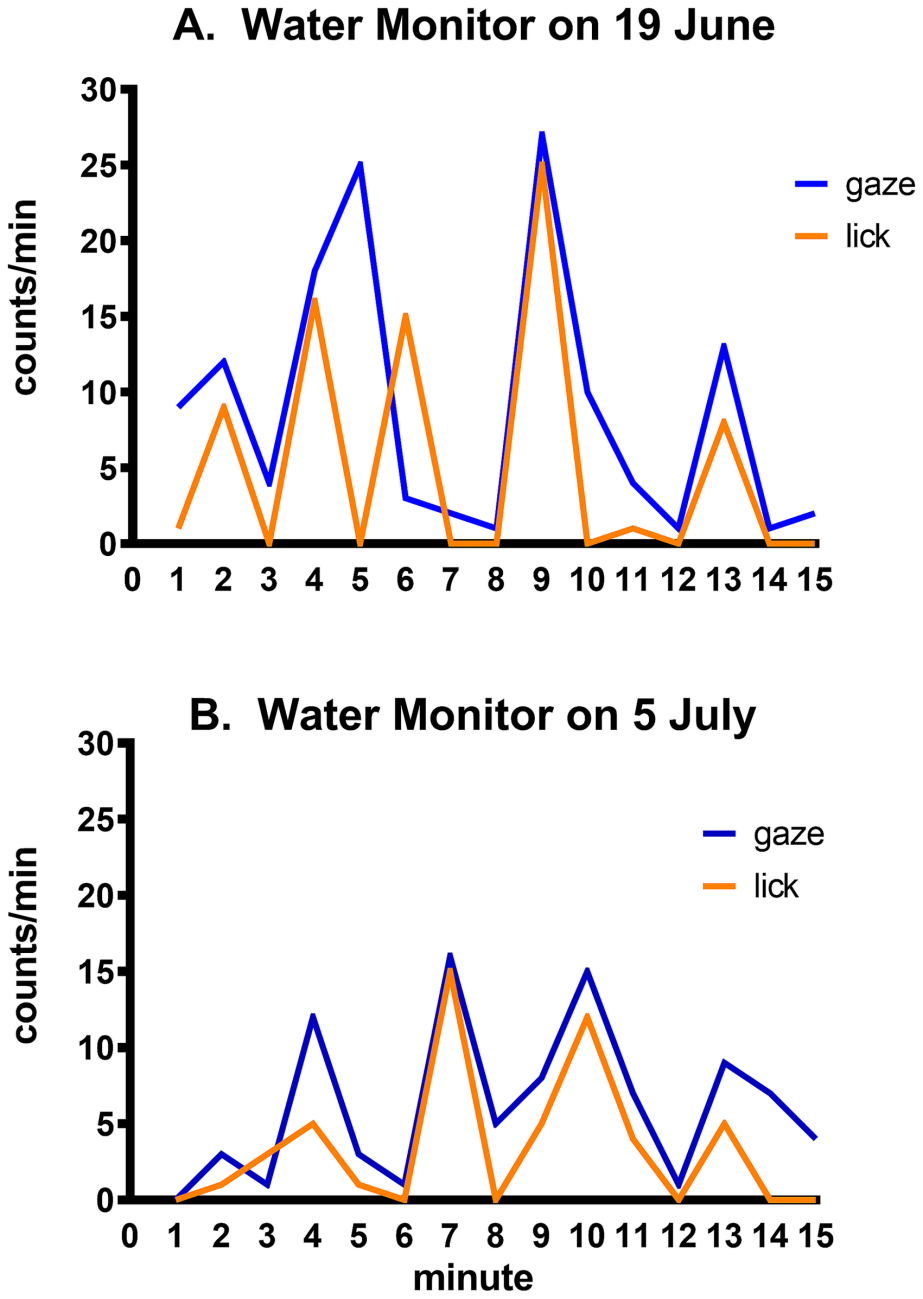
Time course of gaze shifts and tongue licks by the same water monitor on two different days. Minute-to-minute number of gaze shifts and tongue licks (counts/min) by the same lizard in observational sessions on **(A)** 19 June and **(B)** 5 July.

Tongue flicking is an indicator of chemical sensing in lizards. The importance of this mode of environmental monitoring was notably displayed by the water monitor in which the frequency of tongue flicks followed closely the pace of gaze shifts (Figure 5).

**Figure 6:**
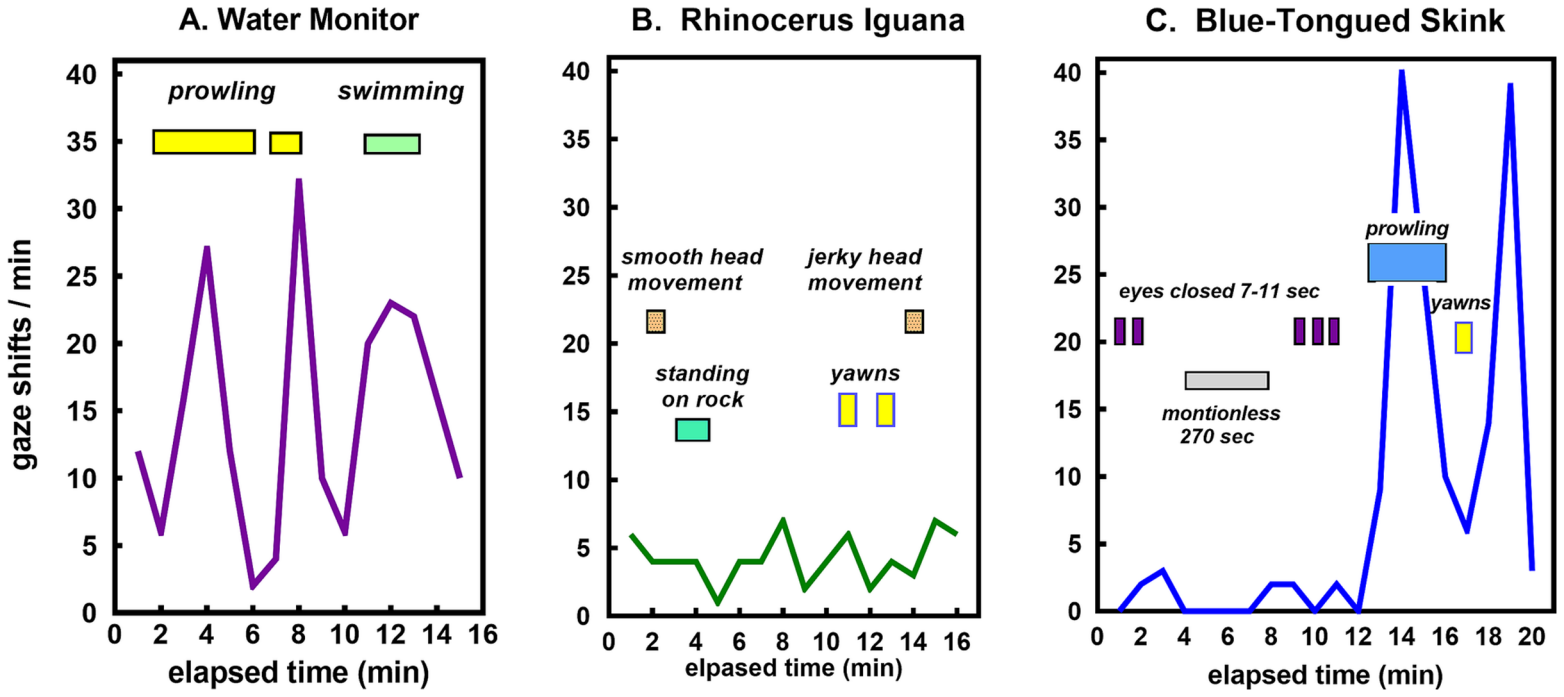
Time course of gaze shifts with corresponding behaviors during a single typical session for the **(A)** water monitor, **(B)** rhinoceros iguana, and **(C)** blue-tongued skink.

Different patterns of sensory experience were illustrated in three different species by discrete behaviors superimposed on the time course of gaze shifts (Figure 6). The water monitor was an active prowler with occasional stops during the first part of the observational session. Toward the end of the session, the monitor dove into the water and swam around for about 3 min before climbing out of the pool (Figure 6A). The iguana was mostly stationary, with a low frequency of gaze shifts, often involving movements of the head but not the body (Figure 6B). The skink was motionless for long periods and seemingly dozing some of the time until late in the session when it started prowling about (Figure 6C).

### 3.2 Birds

Across all birds, the frequency of gaze shifts/min was 55.2 ± 35.3 (mean ± SD), but the tanager was an outlier with 98% probability, so with it excluded, the frequency was 41.9 ± 23.1. This equates to a mean gaze duration of 1.4 s. Thus, the birds shifted their gaze five times faster than the lizards in this study (Figure 2).

A taxonomic breakdown of the bird species observed in at least 3 sessions is given in Figure 3. Gaze shifts for tanagers were more frequent than for the dove (*p* = 0.021) as well as each of the five other species (*p* < 0.0001). The frequency of gaze shifts by the dove was well above that of the other species, but was not a statistically significant outlier from them.

**Figure 7:**
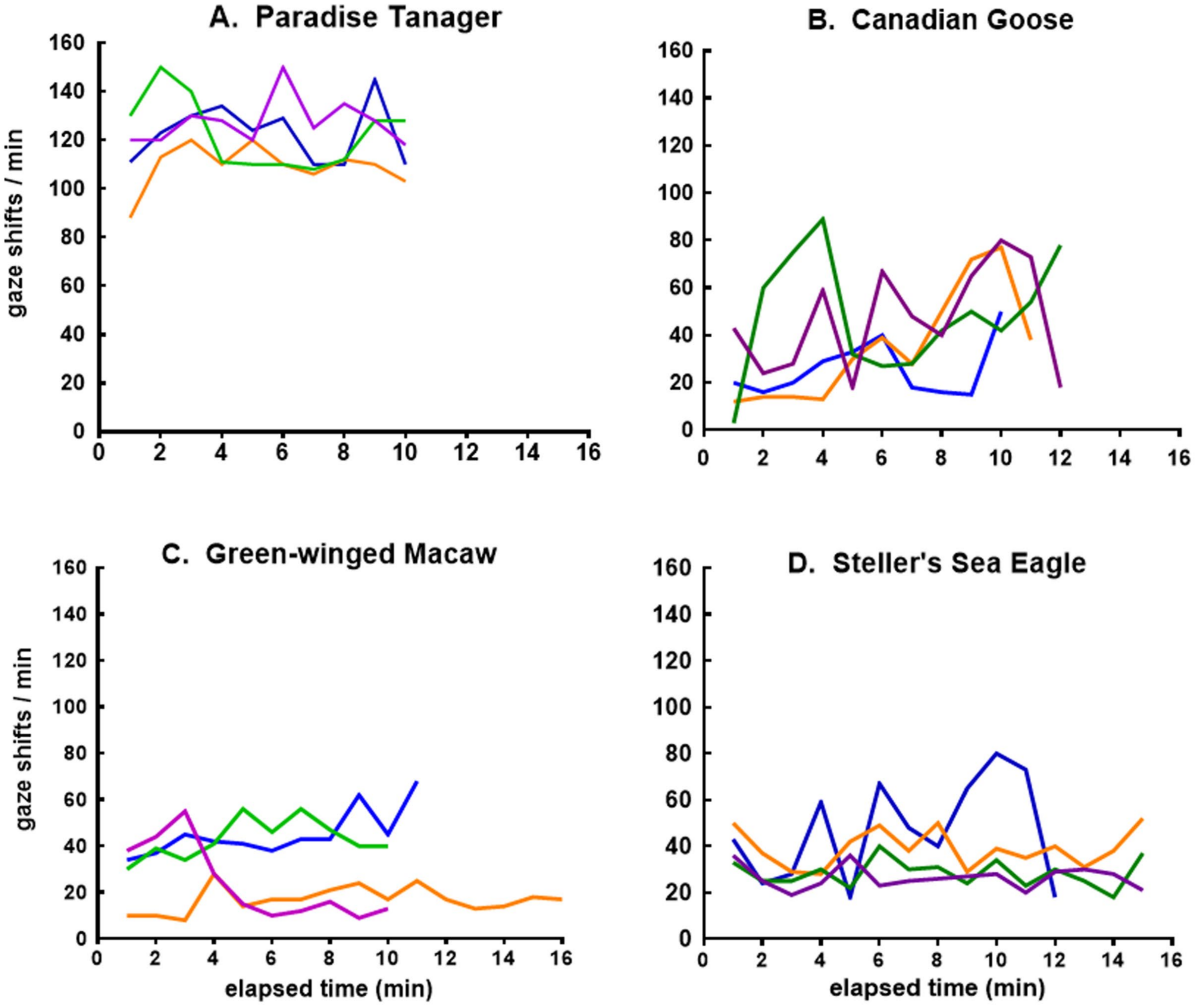
Time course of gaze shifts for the **(A)** tanager, **(B)** goose, **(C)** macaw, and **(D)** eagle. Each line records minute-to-minute rates of gaze shifts on different days by the same eagle and macaw but different tanagers and geese.

The more granular view provided by plots of minute-to-minute variation in the frequency of gaze shifts for four bird species, as for lizards, revealed a qualitatively different pattern for the flow of sensory experience. The tanager showed erratic variations at a high rate of gaze shifts (Figure 7A), while the geese shifted their gaze erratically at a much lower rate (Figure 7B). The macaw shifted its gaze more uniformly over time at a lower rate in some sessions than in others (Figure 7C), while the eagle in 3 out of 4 sessions was relatively uniform and less frequent in its gaze shifts, but showed a more erratic pattern in one session (Figure 7D).

**Figure 8:**
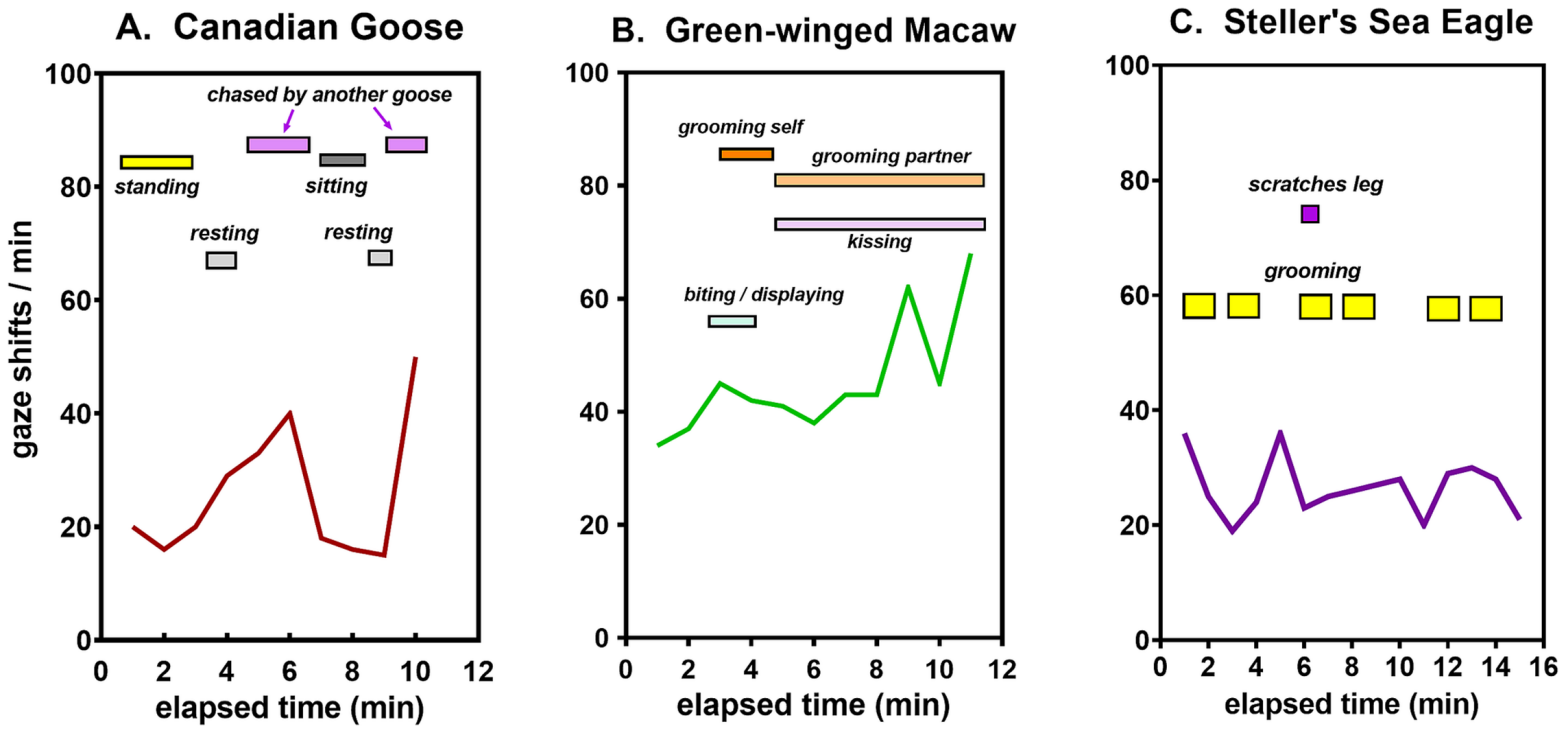
Time course of gaze shifts with corresponding behaviors during a single typical session for a **(A)** goose, **(B)** macaw, and **(C)** eagle.

Different patterns of sensory experience were illustrated in three different birds by discrete behaviors superimposed on the time course of gaze shifts. Throughout all observations on them, geese displayed testy behavior. Typically, they alternated between periods of resting, standing, and interacting with other geese, the frequency of their gaze shifts being correlated with their level of activity (Figure 8A). The macaw generally remained perched in one spot but interacted extensively with a conspecific partner, accounting for its relatively high rate of gaze shifts (Figure 8B). The eagle likewise maintained a stationary perch. Though lacking a conspecific partner, it shifted its gaze frequently in the process of self-grooming and surveying its surroundings (Figure 8C).

## 4 Discussion

The species observed in this study, by virtue of consisting mostly of zoo animals, are skewed toward larger sizes, especially among the birds, so can not be viewed as typical of their respective taxa. Size was not evaluated as an independent variable, but will be pointed out where possibly relevant in the discussion that follows. Predator evasion is an obvious potential corollary of gaze shift frequency (Moore et al., 2017). In this study, only three lizards (the two whiptails and the earless lizard) are subject to significant predation in their natural habitats, while two avian species (tanager and dove) are small enough to be potential prey under field conditions, but systematic correlation of prey evasion tactics with gaze shift frequency was not deemed possible for such a small sample. Where relevant, however, some mention of prey evasion appears in the discussion below.

### 4.1 Lizards

What is it like to be a lizard? It depends.

Once their body temperatures have warmed enough for sufficient neuromuscular function, lizards become active to varying degrees dependent on their size, ecology, diet, and social interactions. Activity patterns fall into three general categories (Pough et al., 1999): sedentary (“sit-and-wait”) predators, cruising foragers, and extensive foragers. The flow of sensory experience varies across these categories, as does the nature of directed attention (Katsuki and Constantinidis, 2014). Sedentary predators rely on ‘bottom-up’ forms of directed attention driven by stimuli salient to the animal’s immediate survival, while extensive foragers appear to make use of a ‘top-down’ strategy based on prior knowledge, willful plans, and current goals. Cruising foragers likely make use of a mixed strategy: ‘top-down’ attention while cruising but ‘bottom-up’ attention when sedentary.

The whiptails and monitors differed from the other lizard species by falling clearly into the active foraging category. Whiptails are small, extremely skittish lizards that forage about almost constantly once they warm up enough to do so (Milstead, 1957; Capula, 1989). Their gaze shifts accordingly are frequent as they search for insects while remaining very wary. The striped whiptail shifted its gaze a mean of 36 times per minute (a mean gaze duration of just 1.7 s). While the frequent and seemingly random gaze shifts help them find food, they also fit the behavioral strategy for successful evasion of predators (Scannell et al., 2001).

The water monitor, like all varanids, is a large carnivorous predator (Zug, 1993). Its combination of frequent gaze shifts and chemical monitoring through tongue licks (Figure 5), and its oscillation between periods of rest and active prowling combined with a transition from terrestrial walking to swimming (Figure 6A), illustrates the variety of sensory information that flows through the course of its daily activities. It has an active purposeful hunting strategy, demonstrating familiarity with the behavioral routines and routes of its prey, indicative of complex behavior and learning (Pough et al., 1999, p. 431) which relies primarily on ‘top-down’ directed attention.

Iguanids like the ones in this study live in warm to hot environments, where they are mostly sedentary during the heat of the day, but move about foraging for insects and patrolling their home ranges in the cooler parts of the morning and afternoon (Irwin, 1965; Baird et al., 1996). As cruising foragers, they presumably make use of both ‘bottom’up’ and ‘top-down’ directed attention. All three iguanids in this study had mean gaze shifts of <12/min, resulting in relatively long mean gaze durations of 5.4 s for the earless lizard, 15.0 s for the rhinocerous iguana, and 26.3 s for the collared lizard. In a single observation of another large, sedentary iguanid, the chukawala had one gaze duration of 107 s. Notably, the earless lizard – the smallest member this group, and the one most vulnerable to predation – had the highest frequency of gaze shifts.

The frilled lizard, in the family Agamidae closely related to the Iguanidae, is similar in size to the rhinocerus iguana and, like most of the iguanids, is primarily an insectivore, but its favored habitat is tree-dwelling rather than ground-dwelling. It too is a sit-and-wait predator with a low mean frequency of 2.6 gaze shifts/min, equating to a gaze duration of 23 s.

The blue-tongued skink is a large lizard distantly related from the iguanids and agamids (Zug, 1993). It lives in grasslands, coastal woodlands, and montane forests, where it stays stationary much of the time, but forages periodically to feed on insects and gastropods which it supplements with berries and flowers (Capula, 1989). As a cruising forager, it too presumably makes use of both ‘bottom’up’ and ‘top-down’ directed attention.

The range of activity patterns among lizards in this study are illustrated by the minute-to-minute changes in gaze shifts shown in Figure 4. The water monitor (Figure 4A), a large active predator, showed the highest rate of gaze shifts. The more passive frilled lizard (Figure 4B) and rhinoceros iguana (Figure 4C) shifted their gaze at consistently low rates, as did the skink most of the time, but during one particular session, it started roaming about actively (Figure 4D).

In summary, what it is like to be a lizard is dictated by diurnal lifestyles constrained by ectothermy, with reliance primarily on diurnal vision and chemical detection. In large tropical species where body temperatures are high enough to sustain activities at any time, some like the water monitor have become active predators using strategies indicative of intelligent planning (Figure 6A). In habitats where high temperatures restrict activity during the heat of the day, many species are relatively inactive for long periods of time, maintaining passive vigilance with infrequent shifts in attention, as in the rhinoceros iguana (Figure 6B). All lizards have to increase their activity episodically for food seeking, courtship, and for many, territorial defense. Some are sit-and-wait insectivores that intersperse long periods of sedentary activity with active foraging. Others oscillate between quiescent periods with little variety in sensory input and episodes of high activity requiring an accelerated rate of sensory processing, as illustrated by the blue-tongued skink (Figure 6C).

### 4.2 Birds

What is it like to be a bird? That too depends on the lifestyle, ecology, and phylogeny of the species under consideration. One clear generalization in contrasting lizards and birds is that the latter as a group change the focus of their attention about five times more frequently than the former (Figure 1), hence their perceptual frames change about five times faster than in lizards.

The extremely high frequency of gaze shifts by the tanager was an outlier from that of the other birds (Figure 3). The paradise tanager flits about actively among the treetops and hops frequently along branches between brief perches, pecking at fruit or poking for insects (Isler and Isler, 1987). As a typical songbird, it relies on keen hearing for social communication. Its mean gaze duration of only 0.49 s was the shortest of any species in this study. The yellow-breasted ground dove is typical of the columbiformes in having a high level of activity, nodding its head with each step as it walks about, pecking at a diet of seeds, grain, fruit, and insects (Peterson, 2010). The other five avian species are all larger birds with lower frequencies of gaze shifts than the dove or tanager. All the birds in this study appear to rely on the ‘top-down’ form of directed attention.

A less obvious difference is that bird behavior tends to be more complex, resulting in gaze durations that vary more within observational sessions and from day to day (Figure 7). Three different patterns of avian social behavior in species with similar gaze shift frequencies are illustrated in Figure 8. The geese were observed walking about in a large group, interacting with one another frequently but never with the same individual for very long (Figure 8A). Two macaws in one typical session maintained a fairly stationary perch, but were in nearly constant interaction with one another, grooming or kissing, or grooming themselves (Figure 8B). Steller’s sea eagle is the largest species of all eagles, with a restricted range along the northern Pacific rim. Like all raptors, it has keen vision for spotting prey from a distance. Even in the breeding season, sea eagles rarely flock together, preferring to perch in isolation. In regions where their diet of salmon or other fish is abundant, they sometimes come together in small groups (Birdfact, 2023), but their social interactions are minimal. Self-grooming is their main activity when not in flight, along with a high frequency of gaze shifts reflecting a constant state of ‘top-down’ directed attention toward their environment (Figure 8C).

In summary, what it is like to be a bird is to rely heavily on vision and, for social communication in some species, sound. Vocalization is well developed and complex in songbirds and some other clades. Like lizards, birds vary in their levels of activity, ranging in this study from the frenetic activity of the tanager to watchful placidity of the eagle. Unlike lizards, birds move about in a three-dimensional world and, perhaps because of that as well as their more complex behaviors and greater tendency to flock together in groups and interact socially, their rate of perceptual framing is roughly five times faster than in lizards.

### 4.3 Lizards v birds

The shortest gaze durations were seen among two of the smallest lizards (striped and checkered whiptail), and the two smallest birds (tanager and dove). But among the remaining lizards and birds, size does not appear to be correlated with gaze duration. If the tanagers and doves in this study are typical of smaller birds in general, the difference in gaze durations between lizards and birds is likely to be even greater than reported here.

A more instructive sense of what it is like to be a lizard or a bird is provided by considering a broader range of life style-parameters that contribute to the subjective experience of the animal, as shown in the contrast of a carnivorous predatory lizard with a carnivorous raptor (Table 1), and a lizard insectivore with an avian vegetarian (Table 2).

**Table 1:** Experience profile for an active lizard and avian predator.

Experience	A. Water monitor	B. Sea eagle
Rate of sensory flow (sec/perceptual frame)		
mean =>	6.5	1.7
maximum =>	>60	3.2
minimum =>	1.9	1.2
Spatial environment (predominantly)	2 dimensional	3 dimensional
Reliance on		
Vision	Significant	Great
Taste	Great	Minimal
Sound	Somewhat	Minimal
Touch	Minimal	Minimal
Social interaction	Minimal	Minimal
Locomotion	Walking & swimming	Perching & flying
Food seeking drive	Strong	Strong

**Table 2:** Experience profile for a low-activity lizard insectivore and avian vegetarian.

Experience	A. Rhinocerus Iguana	B. Macaw
Rate of sensory flow (sec/perceptual frame)		
mean =>	15.0	1.9
maximum =>	>60	7.5
minimum =>	4.0	0.9
Spatial environment (predominantly)	2 dimensional	3 dimensional
Reliance on		
Vision	Great	Great
Taste	Significant	Significant
Sound	Minimal	Significant
Touch	Minimal	Significant
Social interaction	Minimal	Frequent
Locomotion	Walking	Perching & flying
Food seeking	Moderate	Weak

The water monitor is a predatory hunter with a strong drive to seek food after long intervals of not feeding. It stalks its prey with purposeful behavior, guided by visual and chemical sensory input, in essentially a two-dimensional environmental plane. Its gaze duration ranged from 1.9 s during active prowling to more than a minute when it was at rest (Table 1A).

The sea eagle likewise has a strong drive to capture prey over intermittent intervals. It flies through a three-dimensional environment using high-acuity vision to spot, then capture fish. When not in flight it perches, but keeps up a vigilant survey of its surroundings with gaze durations ranging from a mere 1.2 to 3.2 s/perceptual frame (Table 1B).

The rhinoceros iguana is sedentary except when prowling about for insects which are generally abundant in the two-dimensional environment through which the iguana operates. It relies primarily on vision, supplemented by tasting the substrate. Its median gaze duration of 15.5 s is typical for most lizards, except for the frenetic whiptails, and ranges from a minimum of 4 s to longer than a minute. Except for mating and territorial defense, its social interactions are minimal (Table 2A).

The macaw is a highly social animal. In this study it was always observed in nearly constant interaction with a conspecific. It lives in a 3-dimensional environment perching at different heights in trees and flying from one spot to another. Its largely fructivorous diet is readily available in its tropical habitat, minimizing its drive for seeking food (Table 2B). Its mean gaze duration of only 1.9 s is an indication of how fast it processes the flow of visual, auditory, and tactile information, which is devoted mostly to its social interactions.

### 4.4 Summary

The behavioral differences seen in both lizards and birds reflect the differences in lifestyle and environmental constraints that generate unique cognitive requirements for each species (Bräuer et al., 2020; Kaufmann, 2024), consistent with the ecological model of cognition (Gibson, 1979; Rosati, 2017; Irwin, 2024). This study of gaze shifts (and their reciprocal, gaze duration) provides proxy data for the flow of subjective experience. Clearly this is only one of many facets of an animal’s perceptual experience, but in combination with other elements of its behavior, social interaction, and ecological demands, a description of what it is like to be a specific lizard or a specific bird may begin to emerge as pointers toward the nature of that animal’s subjective experience.

Beyond the philosophical sense of what it is like to be a particular animal, behavioral proxies and related indicators of subjective experience can be used as independent variables in studies of neuroanatomical and neurophysiological correlates of animal phenomenology (Sarter et al., 2001; Nani et al., 2019; Ehret and Romand, 2022).

## Data Availability

The original data providing the basis for this study are available from the author upon request.
